# Land Use and Ecosystem Service Value Spatiotemporal Dynamics, Topographic Gradient Effect and Their Driving Factors in Typical Alpine Ecosystems of the East Qinghai‐Tibet Plateau: Implications for Conservation and Development

**DOI:** 10.1002/ece3.71125

**Published:** 2025-03-16

**Authors:** Bin Feng, Jianwei Zhou, Lu Hu, Zherong Liu, Yi Yang, Shuai Yang, Jianying Ni, Wenke Bai, Shanshan Zhao

**Affiliations:** ^1^ Key Laboratory of Southwest China Wildlife Resources Conservation, China West Normal University Nanchong China; ^2^ School of Ecology and Environment, Tibet University Lhasa China; ^3^ Institute of Ecology, China West Normal University Nanchong China; ^4^ Changshagongma National Nature Reserve Service Center China; ^5^ College of Management, China West Normal University Nanchong China

**Keywords:** alpine ecosystem, ecosystem services value, land use, Qinghai‐Tibet Plateau, spatiotemporal evolution

## Abstract

Shiqu County, China, represents a vital component of alpine ecosystems on the eastern Qinghai‐Tibet Plateau, playing a pivotal role in regional ecological functions. However, amid the backdrop of climate change and rapid land‐use transformation, its delicate alpine ecosystems remain persistently threatened by human activities, including several nature reserves and a Ramsar site within it. This paper analyzes the temporal and spatial evolution and differentiation of Ecosystem Service Value (ESV) in Shiqu County (1980–2020), focusing on its driving factors. The research findings reveal that: (1) From 1980 to 2020, Shiqu County experienced a general trend of diminishing unused land, grassland, and cultivated land, alongside an expansion of forested land, water areas, and construction land. (2) Throughout the study duration, the ESV of Shiqu County exhibited a growth trajectory, escalating from 26.180 billion CNY in 1980 to 26.848 billion CNY in 2020, representing a net increase of 2.55%. Grasslands played the most substantial role in contributing to Shiqu's ESV. (3) From 1980 to 2020, all ecosystem service functions exhibited ESV growth, with hydrological regulation and water supply notably increasing by 9.07% and 8.81%, respectively. (4) Land‐use distribution demonstrates significant vertical zonation along gradients of altitude, slope, aspect, and terrain ruggedness, resulting in pronounced vertical variations in ESV, per unit area ESV, and ESV across various ecosystem service functions. (5) The spatial differentiation of ESV in Shiqu County is influenced by both natural and economic factors, with natural factors exerting a more substantial influence on spatial disparities than economic factors. The research provides insights to guide policymakers in prioritizing conservation efforts in fragile alpine ecosystems and optimizing land‐use strategies to achieve sustainable development goals, offering a transferable framework for similar regions globally.

## Graphical abstract

Ecosystem service values (ESV) are a significant measure for assessing the quality of regional ecological environments. Consequently, we analyzed the spatiotemporal dynamics and topographic gradient effect of ESV in the region from 1980 to 2020, employing methodologies such as the ESV assessment model, topographic gradient classification, and geodetector analysis. Additionally, we explored the driving factors behind the ESV. Through a comprehensive analysis of the ecosystem service values in Shiqu County, the research provides in‐depth insights for assessing ecosystem service values in similar regions.

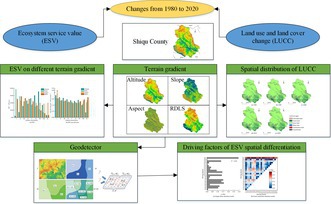

## 1 Introduction

The Qinghai‐Tibet Plateau, often referred to as the “Roof of the World” and the “Asian Water Tower”, occupies a unique and vital position in the global ecological landscape due to its exceptional terrain and intricate ecosystems thriving in its challenging topography (Qiuan et al. 2023; Yu et al. 2023). Alpine ecosystems, which are characteristic of the Qinghai‐Tibet Plateau, are known for their complex diversity and play a crucial role in the region's ecology (Yang et al. 2023a). Alpine ecosystems on the Qinghai‐Tibet Plateau include alpine forests, grasslands, glacial permafrost regions, and wetlands, which together create a diverse and interconnected ecological landscape (Liu et al. 2018; Stokstad 2020; Wei et al. 2021; Yang et al. 2022). Covering about 22% of the Earth's terrestrial surface, alpine ecosystems hold substantial ecological value and support 25% of global biodiversity (Hansen et al. 2013). Globally, approximately 920 million people reside in alpine ecosystems, with 560 million in mountainous regions of China (Yang et al. 2023b). Alpine ecosystems provide a wide array of direct and indirect services to humans, including regulation, support, provision, and cultural services (Yang et al. 2023b), with significant roles in carbon sequestration, water balance, economic stability, and biodiversity preservation (Siles and Margesin 2016). They serve as critical natural reservoirs for carbon sequestration and habitats for various endemic species. However, the eastern Qinghai‐Tibet Plateau is a region sensitive to climate change, having experienced significant warming and increasing humidity over the past two decades (Zhang and Jin 2021). Concurrently, this region also serves as the vanguard of economic development on the Qinghai‐Tibet Plateau, undergoing large‐scale land‐use transformations (Liang and Song 2022). The changes in global climate and the intensification of human developmental activities have exerted immense pressure on regional ecological environments (Zhou et al. 2024). These high‐intensity disturbances have altered the structure and function of regional ecological environments, leading to a drastic increase in the risk of ecological system imbalances. These transformations highlight the pressing necessity for detailed investigations into the spatiotemporal evolution and spatial variations of ecosystem service values, with a particular focus on their implications for sustainable development and ecological resilience in this vulnerable region.

Ecosystem service values quantify the life‐sustaining products and services that humans derive directly or indirectly from the structure, processes, and functions of ecosystems. These ecosystem services include four types of service functions: provisioning, regulating, supporting, and cultural services (Costanza et al. 1997). Ecosystem services underpin human societies and overall welfare (Long et al. 2023) by providing indispensable benefits essential for survival, development, and quality of life (Loomes and O'Neill 1997). Ecosystem service values are a significant measure for assessing the quality of regional ecological environments (Costanza 2020) and serve as an important basis for decisions related to ecological environmental protection, ecological functional zoning, and ecological compensation (Lautenbach et al. 2011). Therefore, comprehending the value and spatio‐temporal distribution of ecosystem services is crucial for informed decision‐making in land management and ecological conservation (Yin et al. 2023). The methodologies used to assess ecosystem services are continuously evolving and diversifying (Li et al. 2023). The approach of quantifying the value of ecosystem services per unit area has been widely adopted in academic research (Costanza et al. 2014). This method allows for the incorporation of the diverse contributions of different land types to ecosystem services, resulting in a more accurate representation of the impacts of land‐use changes on ecosystem services (Yang et al. 2023). In high‐altitude regions, particularly when considering the influence of topography on both land use and ecosystem services, this approach holds significant promise (Cai et al. 2020).

The assessment of ecosystem service values is intricately linked with land use (Chen et al. 2023). Land‐use transformation seeks to balance competing demands from various land‐use types while fulfilling the strategic requirements of ecological civilization, which advocates for harmony between humans and nature through ecological protection, sustainable resource use, and economic‐social integration. Multiple studies have illustrated that unsustainable land‐use practices can have adverse effects on ecosystem services, resulting in land degradation, soil erosion, environmental deterioration, and a chain of interconnected challenges (Lou et al. 2022; Qiu et al. 2021; Rimal et al. 2019; Su et al. 2023). For example, a decline in provisioning services such as agricultural productivity could directly affect livelihoods, while alterations in regulating services like water purification or flood control could increase vulnerability to natural disasters (Veerkamp et al. 2024). To advance ecological conservation and sustainable development strategies, it is essential to comprehend the temporal evolution and spatial disparities in land use and ecosystem service values within alpine ecosystems. Conducting a comprehensive assessment of the relationships, mechanisms, and driving factors linking land use changes with ecosystem service values is crucial. Such an assessment is vital for ensuring sustainable land resource utilization and optimizing ecosystem service functionality (Su et al. 2023).

**FIGURE 1:**
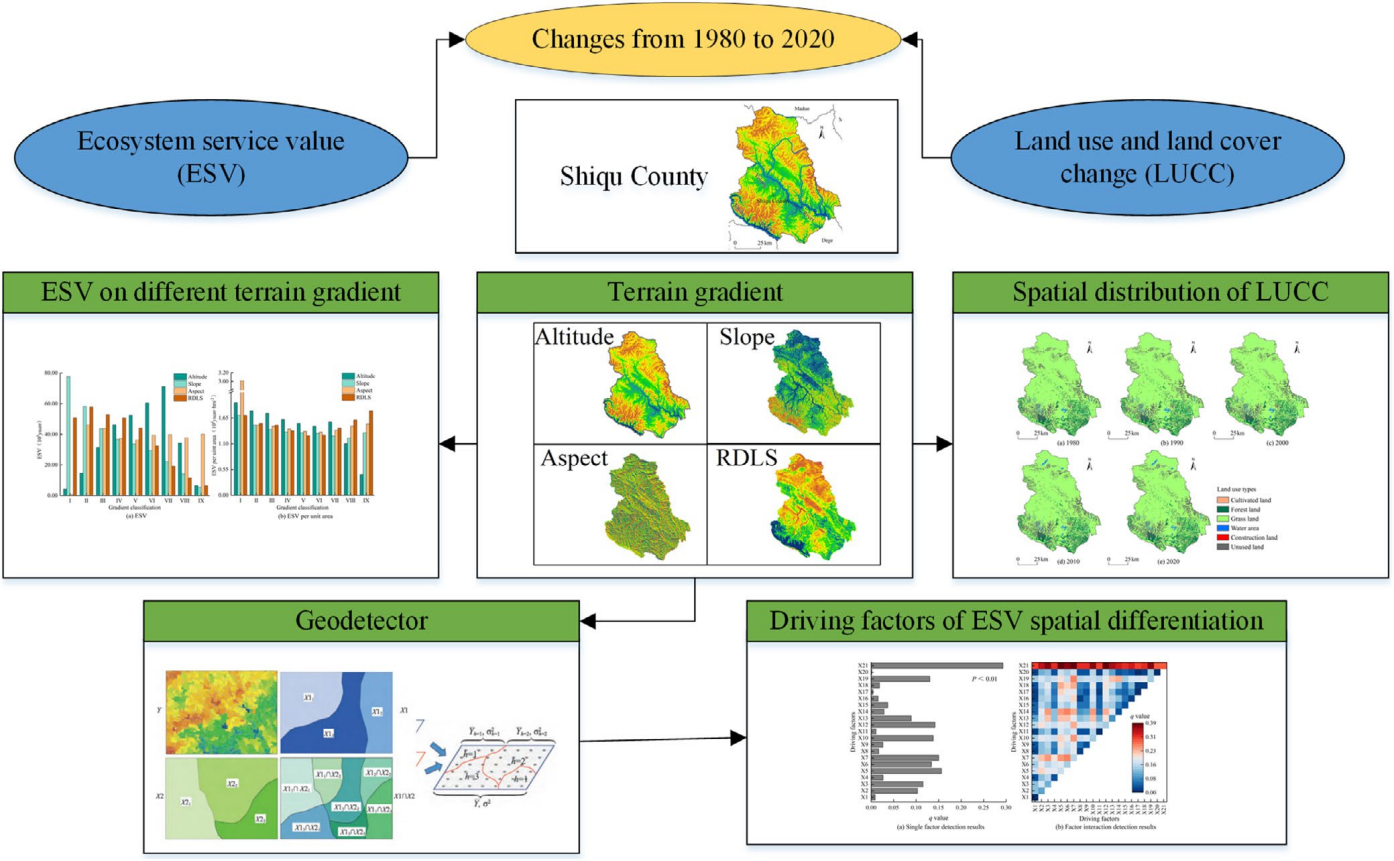
Graphical abstract. Analyzing land‐use changes and ecosystem service value (ESV) in Shiqu County (1980–2020) reveals spatial differentiation influenced by terrain gradients and driving factors.

Previous studies on the Eastern Qinghai‐Tibet Plateau have focused on biodiversity conservation, grassland management, and climate change impacts, but research explicitly linking ESV dynamics with terrain gradient effects and their implications for sustainable development remains limited (Ji et al. 2023; Liu et al. 2024; Xu et al. 2019). While rooted in the Eastern Qinghai‐Tibet Plateau, our findings present a transferable framework for analyzing ESV dynamics and integrating ecological protection with socioeconomic development in fragile ecosystems globally. In the unique context of the Eastern Qinghai‐Tibet Plateau, there is a compelling need for a thorough exploration of the spatiotemporal evolution and spatial differentiation of ecosystem service values. This exploration will provide valuable insights to guide land management decisions and policy formulation. We utilized a dataset spanning eight time periods from 1980 to 2020 for Shiqu County, employing methodologies such as the ESV assessment model, topographic gradient classification, and geodetector analysis to conduct a comprehensive evaluation of ecosystem service values and explore the driving factors behind the ESV. This study focuses on examining the effects of terrain gradients on ESV dynamics in the Eastern Qinghai‐Tibet Plateau and identifying the primary factors influencing spatial variations (Figure 1). Through a comprehensive analysis of the ecosystem service values in Shiqu County, the research provides in‐depth insights for assessing ecosystem service values in similar regions. This information is intended to guide forthcoming land management and ecological conservation policies, further advancing the overarching goals of sustainable development and ecological protection.

## 2 Materials and Methods

### 2.1 Study Area

**FIGURE 2:**
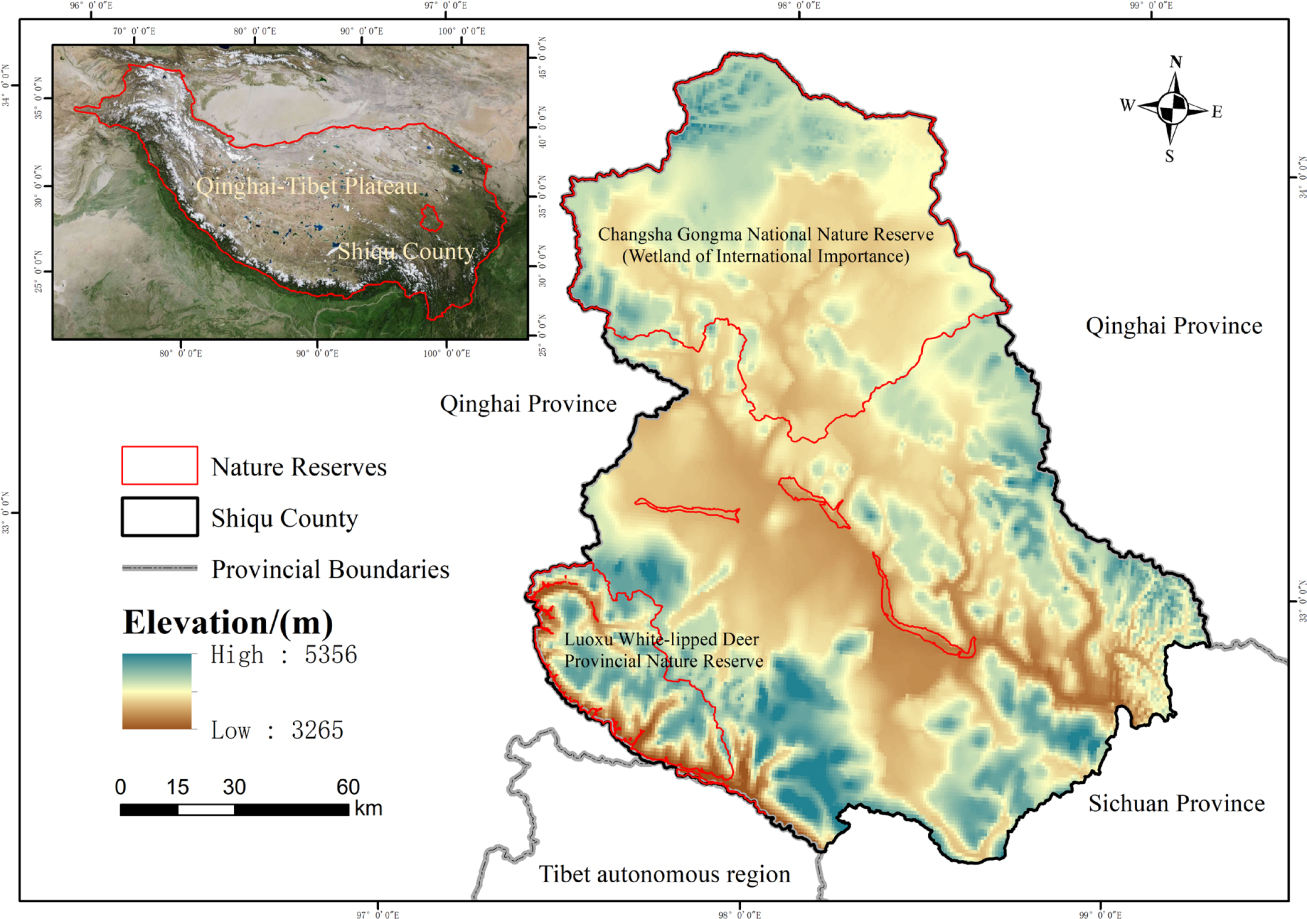
Study area. The location of Shiqu County on the Qinghai‐Tibet Plateau, its elevation range, and the distribution of nature reserves within the region.

Shiqu County is located at the eastern Qinghai‐Tibet Plateau, at the tri‐junction of Sichuan, Qinghai, and Tibet provinces in China (Figure  2). Spanning an area of 25,191 km^2^, Shiqu County is the largest and highest county in Sichuan Province, governing 22 townships with a population of 101,000 (Hao et al. 2020). The county falls within the North Asian cold temperate climate zone, with an average annual temperature of −0.9°C. It experiences annual sunshine hours ranging from 2410 to 2530 h, with a sunshine percentage of 55%–57%, and the total annual solar radiation is between 140–170 kc/m. The average annual precipitation is 569.6 mm (Wang et al. 2020). Shiqu County boasts the most typical alpine ecosystem on the eastern Qinghai‐Tibet Plateau, with altitudes ranging from 3214 to 5298 m and an average elevation of 4526 m. The ecosystem types are diverse, including forests covering 2044.38 km^2^, grasslands spanning 15,149.22 km^2^, and wetlands of 3984.78 km^2^ (Derived from the Forestry and Grassland Bureau of Shiqu County). Additionally, Shiqu County holds significant ecological value. In the north, it is home to the Changshagongma National Nature Reserve (Ramsar site), predominantly characterized by wetlands and alpine grasslands, covering an area of 6698.00 km^2^. In the south lies the Luoxu Provincial Nature Reserve, dominated by alpine forests, spanning 1553.50 km^2^. As a typical representative of the alpine ecosystems at the eastern Qinghai‐Tibet Plateau, exploring the spatiotemporal variations and spatial differentiation characteristics of the ecosystem service value in Shiqu during periods of rapid economic development and swift changes in land use holds significant value for guiding land management decisions and policy formulation on the Qinghai‐Tibet Plateau.

### 2.2 Data Sources

The study utilized land‐use grid data for the years 1980, 1990, 2000, 2010, and 2018, sourced from the Chinese Academy of Sciences Resource and Environment Science and Data Center (http://www.resdc.cn/). This dataset, derived from the interpretation of Landsat remote sensing imagery, has a resolution of 30 m with an overall accuracy exceeding 90% (Liu et al. 2014). Land‐use types were classified into six categories according to the national land‐use remote sensing monitoring classification system: cultivated land, forest land, grassland, water areas, construction land, and unused land. Data on annual average wind speed, annual sunshine hours, annual average relative humidity, annual average ground temperature, annual evaporation, annual average precipitation, annual average temperature, NDVI, accumulated temperature ≥ 10°C, per capita GDP, and population density were also obtained from the Chinese Academy of Sciences Resource and Environment Science Data Center (http://www.resdc.cn/), with a resolution of 1 km. Slope, aspect, and terrain ruggedness, with a resolution of 30 m, were derived from DEM data sourced from the Geospatial Data Cloud (http://www.gscloud.cn/). Road, settlement, river, lake, and reservoir data were obtained from the National Basic Geographic Information Center (https://www.webmap.cn/).

### 2.3 Research Methodology

#### 2.3.1 Ecosystem Service Value Assessment Model

Based on the ESV (Ecosystem Service Value) assessment model (Costanza et al. 1997) (Formula 1), and referring to the unit area ESV equivalent table for terrestrial ecosystems in China (Chen and Zhang 2000), the study calculates the ESV for Shiqu County. This calculation is based on the average grain yield of Shiqu County (2701.51 kg/ha) and the 2020 procurement prices for major crops in Sichuan Province (2.41 CNY/kg). Following the principle that “the unit area ESV is equal to one‐seventh of the market economic value of the average grain yield per unit area” (Xie et al. 2015), the ESV value equivalent for Shiqu County is determined to be 930.09 CNY/ha. Subsequently, the ESV of the study area is calculated using the equivalent table (Table 1) with the following formula:

(1) $$\text{ESV}=\sum_{i=1}^{m}\sum_{j=1}^{n}A_{i}\times S_{\mathrm{ij}}$$

**TABLE 1:** The equivalent of ecological service value per unit area in Shiqu County (CNY/ha).

Ecosystem service	Land‐use types					
	Cultivated land	Forest land	Grassland	Water area	Construction land	Unused land
FP	1264.92	288.33	213.92	744.07	9.30	158.12
RMP	372.04	660.36	316.23	213.92	0.00	27.90
WRS	18.60	344.13	176.72	7710.46	0.00	18.60
GR	1032.40	2185.71	1125.41	716.17	0.00	102.31
CR	530.15	6538.54	2966.99	2129.91	0.00	93.01
EP	158.12	1850.88	976.60	5162.01	0.00	288.33
WR	2529.85	3264.62	2176.41	95092.53	0.00	195.32
SC	9.30	2660.06	1367.23	864.98	18.60	120.91
NCM	176.72	204.62	102.31	65.11	0.00	9.30
BM	195.32	2418.24	1246.32	2371.73	316.23	111.61
AL	83.71	1060.30	548.75	1757.87	9.30	46.50
TL	6371.13	21475.81	11216.90	116828.77	353.43	1171.92

Abbreviations: AL: Esthetic landscape; BM: Biodiversity maintenance; CR: Climate regulation; EP: Environment purification; FP: Food production; GR: Gas regulation; NCM: Nutrient cycle maintenance; RMP: Raw material production; SC: Soil conservation; WR: Water regulation; WRS: Water resource supply; TL: Total. The same below.

In the formula, ESV represents the ecosystem service value. *A*_*i*_ represents the area of land category *i* in the evaluation price network. *S*_*ij*_ is the ESV equivalent per unit area of *j* ecosystem service types for *i* land types. i is the number of land types. *j* represents the type of ecosystem service.

#### 2.3.2 Sensitivity Analysis

The sensitivity index can be used to verify the extent to which changes in ESV over time depend on fixed value coefficients (VC), thus reducing uncertainty in the results (Wang et al. 2022). Relevant scholars typically calculate the sensitivity index by increasing or decreasing the ecosystem service value coefficients for each land‐use type by 50% (Guo et al. 2022). The calculation formula is as follows:

(2) $$\text{CS}=\left|\frac{\left({\text{ESV}}_{j}-{\text{ESV}}_{i}\right)/{\text{ESV}}_{i}}{\left({\text{VC}}_{\text{jk}}-{\text{VC}}_{\text{ik}}\right)/{\text{VC}}_{\text{ik}}}\right|$$

In the formula, *VC*_*ik*_ and *VC*_*jk*_ represent the ecosystem service value coefficients per unit area for ecosystem type *k* before and after adjustment, respectively; ESV_*i*_ and ESV_*j*_' represent the total ecosystem service values before and after adjustment, respectively. (ESV_*j*_ − ESV_*i*_)/ESV_*i*_ denotes the rate of change in ecosystem service value, and (*VC*_*jk*_ − *VC*_*ik*_)/*VC*_*ik*_ represents the rate of change in the value coefficient. *CS* is the sensitivity index for the ecosystem service value coefficients in the study area. If *CS* > 1, it indicates that ESV is highly elastic to *VC*, meaning that the accuracy and reliability of the value coefficient are relatively low. Conversely, if *CS* < 1, it indicates that ESV is inelastic to *VC*, making the results more reliable.

#### 2.3.3 Terrain Factors Extraction

Select four terrain elements: altitude, slope, aspect, and terrain undulation to analyze the terrain gradient distribution of land use and ESV in Shiqu County. The elevation and slope are classified using the natural breakpoint method (Jin et al. 2023) (Table 2), and the aspect is classified based on previous research results (Fu et al. 2021). The terrain undulation is determined by the elevation difference between the highest and lowest points in a specific area (Zhang and You 2013).

**TABLE 2:** Gradient classification criteria of terrain factors in Shiqu County.

Classification	Altitude (m)/proportion (%)	Slope (°)/ proportion (%)	RDLS (m)/ proportion (%)	Aspect/proportion (%)
I	[3214,3723]/0.96	[0,4.08]/20.35	[0,110]/13.33	Plain/0.13
II	(3723,4034]/3.66	(4.08,7.66]/17.34	(110,192]/16.89	North/13.76
III	(4334,4199]/8.05	(7.66,11.52]/13.95	(192,273]/15.75	Northeast/13.32
IV	(4199,4337]/12.78	(11.52,15.56]/12.18	(273,351]/16.50	East/11.77
V	(4337,4465]/15.35	(15.56,19.70]/11.28	(351,429]/15.49	Southeast/11.92
VI	(4465,4586]/18.40	(19.70,24.07]/10.00	(429,519]/11.30	South/13.07
VII	(4586,4708]/20.32	(24.07,29.00]/7.88	(519,642]/5.95	Southwest/12.80
VII	(4708,4855]/13.95	(29.00,35.30]/5.18	(642,809]/3.21	West/11.45
IX	(4855,5298]/6.53	(35.30,72.91]/1.85	(809,1261]/1.59	Northwest/11.77

#### 2.3.4 Driver Factors Analysis

Geodetector analysis is a statistical method used to detect and quantify the influence of explanatory variables on spatially stratified heterogeneity. It evaluates the extent to which a particular factor explains the spatial distribution of a dependent variable, offering insights into the driving forces behind observed patterns. Geodetector can explore the spatial differentiation characteristics of elements (Wang et al. 2010) and use factor detectors and interaction detectors to detect the driving factors and their interactions of ESV spatial differentiation in Shiqu County. The formula is as follows:

(3) $$q=1-\frac{1}{N\sigma^{2}}\sum_{h=1}^{L}N_{h}\sigma_{h}^{2}$$

In the formula, *q* is the influence of the driving factor on the spatial differentiation of ESV; *h* is the stratification/partitioning of variable Y or factor X; $\sigma_{h}^{2}$ and $\sigma^{2}$ are the variance of the evaluation unit and the Y value of the entire region, respectively; *N*_*h*_ and *N* represent the number of units in the *h* area and the entire area, respectively.

#### 2.3.5 Standard Deviation Elliptic Theory

The standard deviational ellipse method is a spatial analysis technique that reflects the spatial distribution characteristics and spatiotemporal evolution of the study object by describing fundamental parameters such as centroid, azimuth, major axis, and minor axis (Zhao et al. 2022). This method is used in this study to analyze the spatiotemporal distribution and evolution characteristics of ESV. The formulas for calculating the key parameters are as follows (Du et al. 2019).

(4) $$\bar{X}=\frac{\sum_{i=1}^{n}w_{i}x_{i}}{\sum_{i=1}^{n}w_{i}},\bar{Y}=\frac{\sum_{i=1}^{n}w_{i}y_{i}}{\sum_{i=1}^{n}w_{i}}$$

(5) $$\tan\alpha =\frac{\left(\sum_{i=1}^{n}{w_{i}}^{2}{{\bar{x}}_{i}}^{2}-\sum_{i=1}^{n}{w_{i}}^{2}{{\bar{y}}_{i}}^{2}\right)+\sqrt{{\left(\sum_{i=1}^{n}{w_{i}}^{2}{{\bar{x}}_{i}}^{2}-\sum_{i=1}^{n}{w_{i}}^{2}{{\bar{y}}_{i}}^{2}\right)}^{2}+4\sum_{i=1}^{n}{w_{i}}^{2}{{\bar{x}}_{i}}^{2}{{\bar{y}}_{i}}^{2}}}{2\sum_{i=1}^{n}{w_{i}}^{2}{{\bar{x}}_{i}}^{2}{{\bar{y}}_{i}}^{2}}$$

(6) $$\sigma_{x}=\sqrt{\frac{\sum_{i=1}^{n}{\left(w_{i}{\bar{x}}_{i}\cos\alpha -w_{i}{\bar{y}}_{i}\sin\alpha \right)}^{2}}{\sum_{i=1}^{n}{w_{i}}^{2}}},\sigma_{y}=\sqrt{\frac{\sum_{i=1}^{n}{\left(w_{i}{\bar{x}}_{i}\sin\alpha -w_{i}{\bar{y}}_{i}\cos\alpha \right)}^{2}}{\sum_{i=1}^{n}{w_{i}}^{2}}}$$

(7) $$S=\pi \sigma_{x}\sigma_{y}$$

In formulas ((4), (5), (6), (7)), $\bar{x}$ and $\bar{y}$ represent the coordinates of the centroid; *W*_*i*_ is the weight; α is the azimuth of the standard deviational ellipse; ${\bar{x}}_{i}$ and ${\bar{y}}_{i}$ are the deviations of the coordinates of each study object from the mean center; *σ*_*x*_ and *σ*_*y*_ represent the standard deviations along the *x*‐axis and *y*‐axis, respectively; and *S* denotes the area of the ellipse.

## 3 Results

### 3.1 Spatiotemporal Changes in Land Use

**FIGURE 3:**
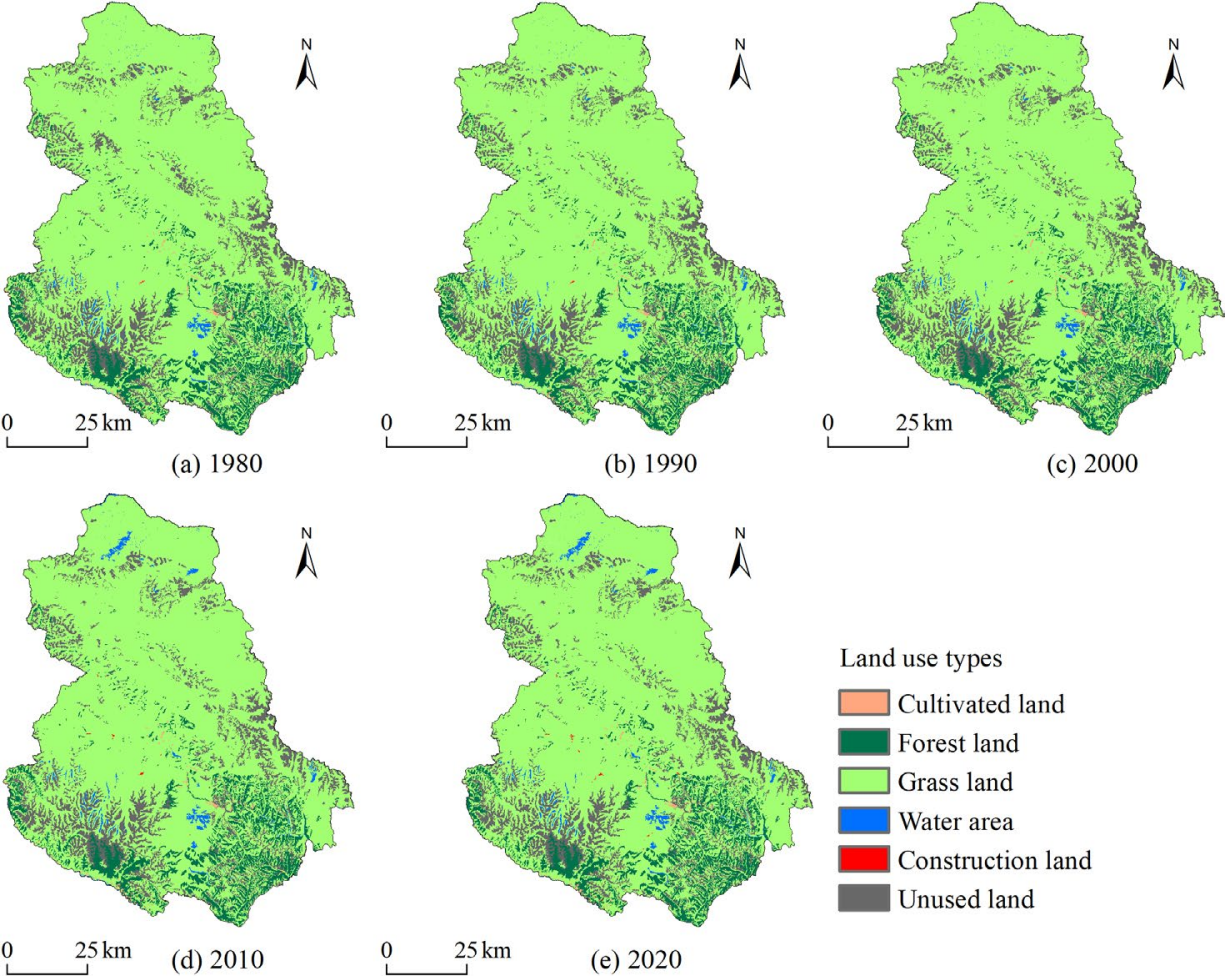
Spatial distribution of land use in Shiqu County from 1980 to 2020. (a) 1980 (b) 1990 (c) 2000 (d) 2010 (e) 2020.

Shiqu County's land use is primarily dominated by grasslands and unused lands (Figure 3), accounting for approximately 77.96% and 12.15% of the total county area, respectively. Grasslands are widely distributed in the central and northern regions, while unused lands are concentrated in the alpine zones of the southeast and southwest, as well as in the northern mountainous areas. Following these are forest lands, which constitute about 8.70% of the total area, predominantly located in the southern mountains. Additionally, the areas of cultivated land, water areas, and construction land each make up less than 1.00% of the total area, with cultivated and construction lands primarily located in the Yalong River valley, and water areas mainly found in the southern alpine lakes, the main tributaries of the Yalong River, and the northern wetland areas.

**FIGURE 4:**
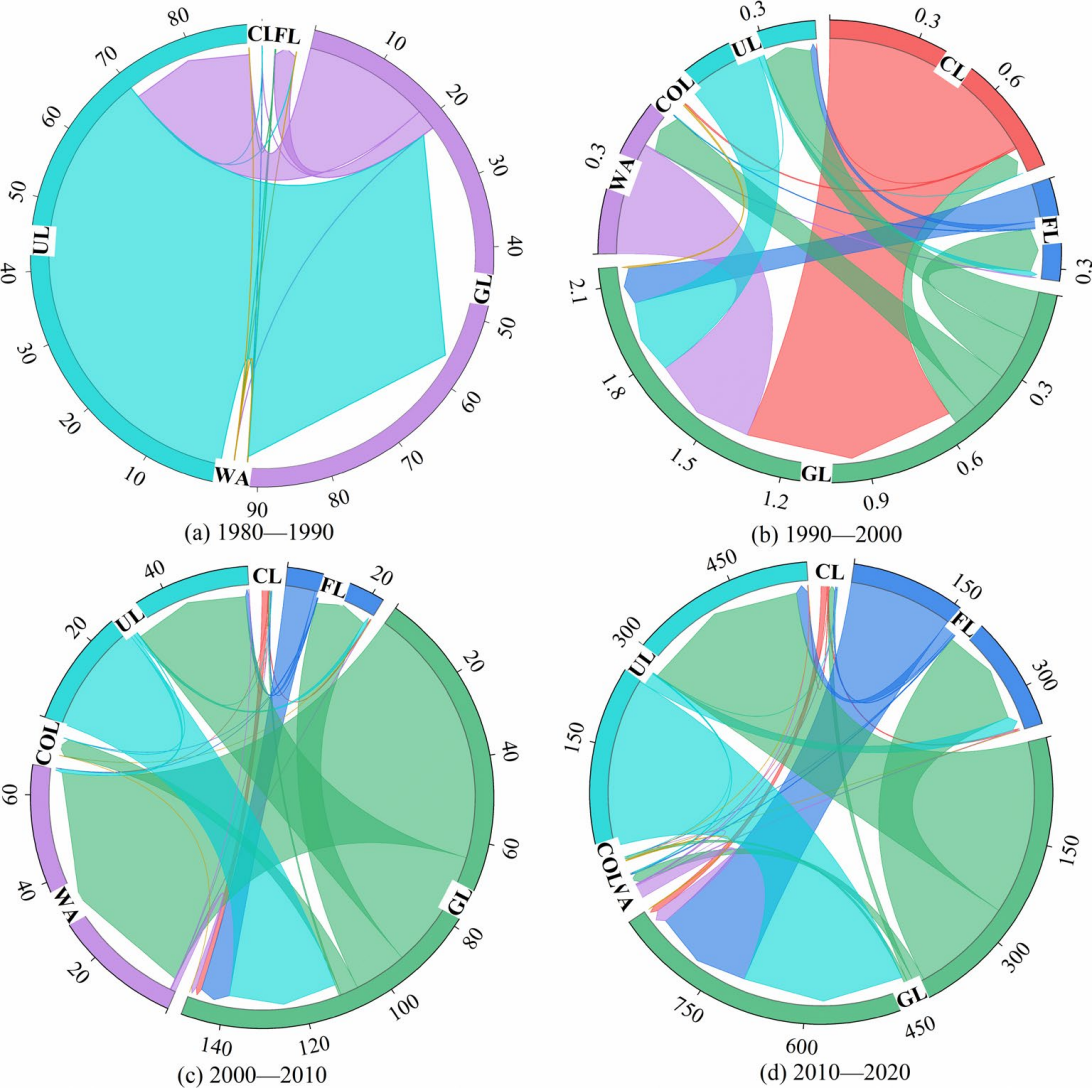
Land‐use changes in Shiqu County from 1980 to 2020. (a) 1980–1990, (b) 1990–2000, (c) 2000–2010, and (d) 2010–2020. CL: Cultivated land; FL: Forest land; GL: Grassland; WA: Water area; COL: Construction land; UL: Unused land.

From 1980 to 2020, the overall trend in land use was a reduction in unused land, grassland, and cultivated land, and an expansion of forest land, water areas, and construction land (Figure 4). Notably, the changes in the areas of construction land, water areas, and cultivated land were particularly significant, with change rates of 344.10% (8.32 km^2^), 33.53% (59.75 km^2^), and − 2.42% (1.49 km^2^), respectively. Additionally, the change rates of forest land, grassland, and unused land were 0.41% (8.01 km^2^), −0.21% (35.95 km^2^), and − 1.95% (53.70 km^2^), respectively.

### 3.2 Sensitivity Analysis of Ecosystem Service Values

The value coefficients for cropland, forestland, grassland, water bodies, construction land, and unused land were each adjusted upward by 50%, and the ESV sensitivity index for Shiqu County from 1980 to 2020 was calculated (Table 3). The results show that the sensitivity indices for all land‐use types in the study area were below 1. Among them, the sensitivity index *CS* for construction land was the lowest, with a value close to 0. This indicates that a 1% increase in the area of construction land *VC* would result in almost no increase in ESV, suggesting that the accuracy of the value coefficient for construction land has minimal impact on the total ESV. The sensitivity indices *CS* for cropland, forestland, water bodies, and unused land were also all below 0.2. Grassland, in particular, covers a vast area in Shiqu County and has a relatively high value coefficient, making its sensitivity index the highest. From 1980 to 2020, the sensitivity index *CS* for grassland consistently exceeded 0.72, indicating that for every 1% increase in grassland area *VC*, the total ESV of the study area would increase by more than 0.72%. Overall, the total ESV of the study area is inelastic, demonstrating that the ecosystem service value coefficients, adjusted to reflect the regional characteristics of Shiqu County, are reasonable, and the study results are reliable.

**TABLE 3:** Sensitivity index of ESV in Shiqu County from 1980 to 2020.

Land‐use type	Year				
	1980	1990	2000	2010	2020
Cultivated land	0.001500	0.001497	0.001480	0.001419	0.001427
Forest lands	0.159335	0.159244	0.159259	0.155709	0.156011
Grasslands	0.747286	0.747798	0.747907	0.725996	0.727190
Water areas	0.079520	0.079359	0.079252	0.105068	0.103544
Construction land	0.000003	0.000003	0.000003	0.000009	0.000014
Unused lands	0.012356	0.012099	0.012100	0.011799	0.011814

### 3.3 Temporal and Spatial Variation of Ecosystem Service Values

**FIGURE 5:**
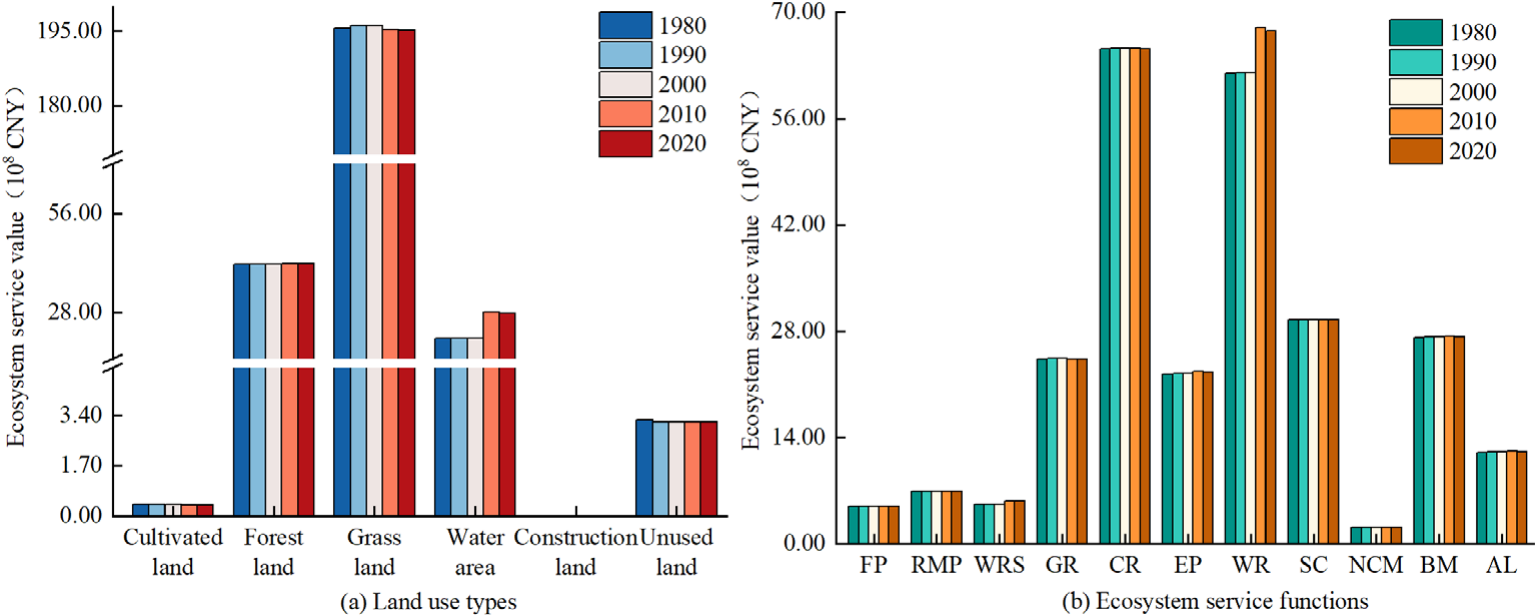
ESV changes in Shiqu County from 1980 to 2020: (a) ESV of various land‐use types from 1980 to 2020, and (b) ESV of different ecosystem service functions from 1980 to 2020.FP: Food production; RMP: Raw material production; WRS: Water resource supply; GR: Gas regulation; CR: Climate regulation; EP: Environment purification; WR: Water regulation; SC: Soil conservation; NCM: Nutrient cycle maintenance; BM: Biodiversity maintenance; AL: Esthetic landscape; TL: Total. The same below.

During the study period, the overall Ecosystem Service Value (ESV) of Shiqu County exhibited a growth trend, increasing from 26.180 billion CNY in 1980 to 26.848 billion CNY, a net increase of 2.55% (0.668 billion CNY). Analyzing the ESV contributions of various land uses (Figure 5a), grasslands were the largest contributor to Shiqu's ESV, accounting for approximately 73.92%, followed by forest land (15.79%) and water areas (8.93%), while the ESV contribution rates of cultivated land, construction land, and unused land were all less than 1.50%. From 1980 to 2020, the ESV changes of each land‐use type were consistent with their area changes, among which the ESV growth of water areas was particularly significant, increasing by 33.53% (0.841 billion CNY).

Examining the ESV contributions of various ecosystem service functions (Figure 5b), climate regulation and hydrological regulation stood out, accounting for approximately 24.63% and 24.28% of the total ESV, respectively, followed by soil retention (11.16%), biodiversity (10.29%), and gas regulation (9.21%), with the contribution rates of other ecosystem service functions below 9.00%. From 1980 to 2020, the ESV of all ecosystem service functions showed an increasing trend, particularly in hydrological regulation and water supply, which increased by 9.07% (0.562 billion CNY) and 8.81% (0.046 billion CNY), respectively. Evidently, the increase in ESV in Shiqu County was mainly due to the enhanced hydrological regulation and water supply capabilities brought about by the expansion of water body areas.

### 3.4 Temporal and Spatial Distribution and Variation of per‐Unit Area ESV

**FIGURE 6:**
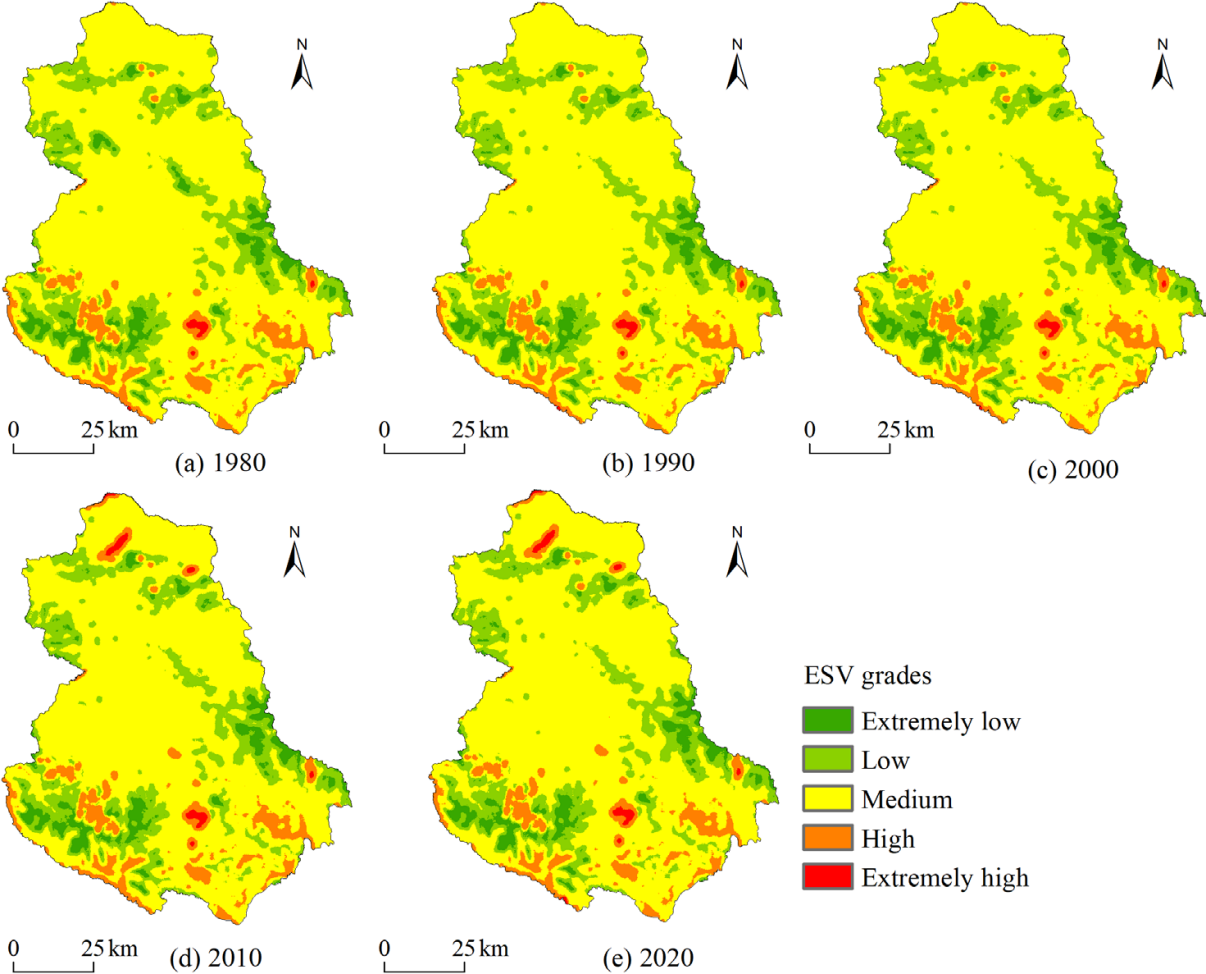
Spatial distribution of ESV at different levels in Shiqu County from 1980 to 2020. (a) 1980 (b) 1990 (c) 2000 (d) 2010 (e) 2020.

**FIGURE 7:**
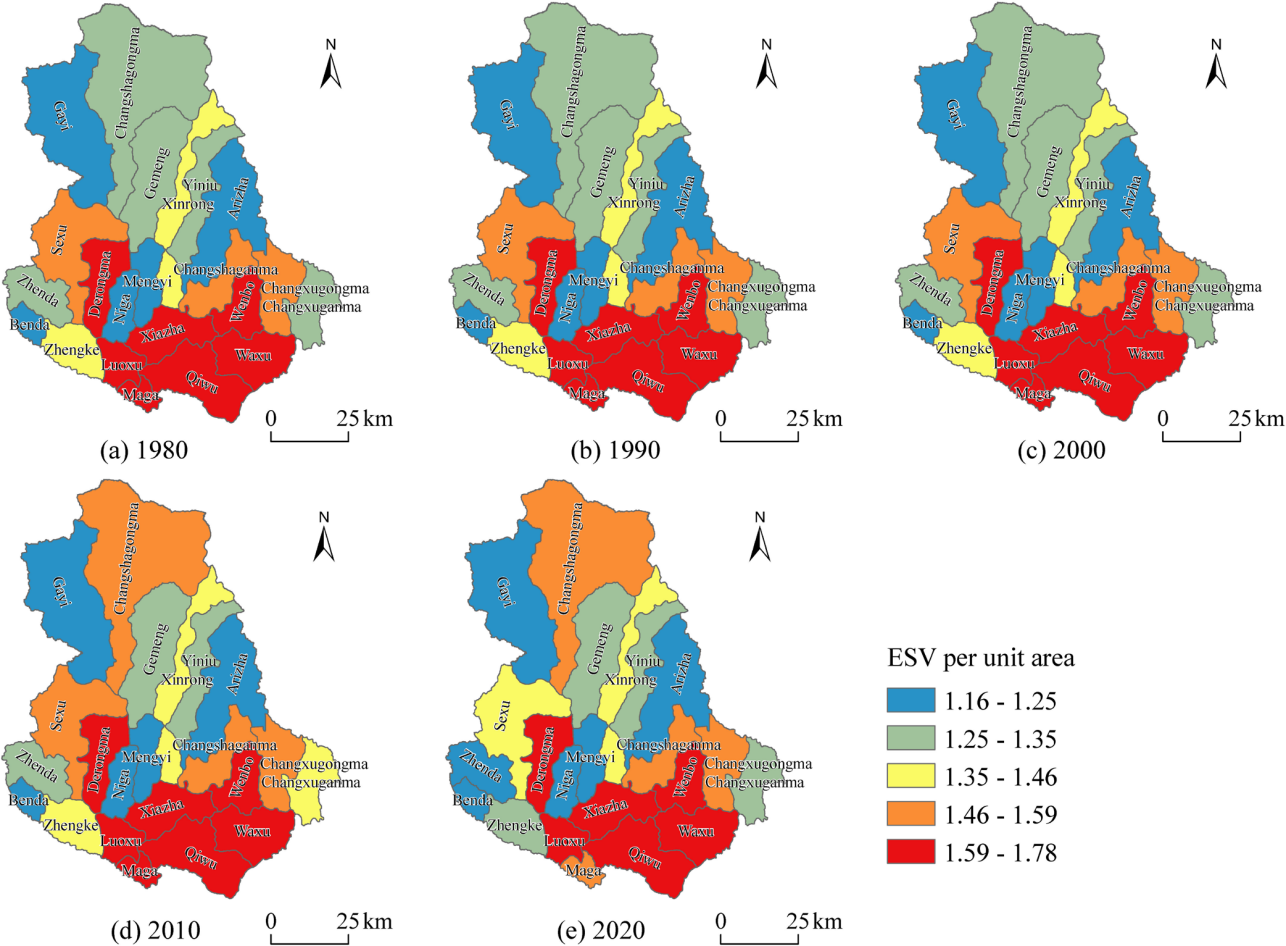
Distribution and changes of ESV per unit area at the township level from 1980 to 2020. (a) 1980 (b) 1990 (c) 2000 (d) 2010 (e) 2020.

To further analyze the spatial distribution and change characteristics of ESV in Shiqu County from 1980 to 2020, the per unit area ESV (10^4^ CNY/ha) was calculated using a 1 km × 1 km valuation grid and at the township scale. The per unit area ESV for the 1 km evaluation units was classified into five categories using the natural breaks method: extremely low [0.15, 0.78), low [0.78, 1.18), medium [1.18, 2.02), high [2.02, 6.14), and extremely high [6.14, 11.41] (Figure 6). The per‐unit area ESV at the township scale was also graded using the natural breaks method (Figure 7).

From the perspective of the global Moran's *I* index of per unit area ESV (Table 4): at the scale of 1 km × 1 km evaluation units, per unit area ESV shows significant spatial positive correlation, and its spatial positive correlation overall tends to weaken from 1980 to 2020. This is evidenced by a decrease in Moran's *I* from 0.484 in 1980 to 0.474 in 2020, with the highest value occurring in 2010 (Moran's *I* = 0.580). At the township scale, there is no significant spatial autocorrelation in per unit area ESV (*p* > 0.05, |Z| < 2.58).

**TABLE 4:** Changes in the Global Moran Index of unit area ESV at the 1 km evaluation unit and township scale.

Scale	Global Moran Index	Years				
		1980	1990	2000	2010	2020
1 km × 1 km	Moran's *I*	0.484	0.483	0.483	0.580	0.474
	*Z*	103.188	102.904	102.969	123.276	103.589
	*P*	< 0.001	< 0.001	< 0.001	< 0.001	< 0.001
Township	Moran's *I*	0.230	0.225	0.225	0.179	0.167
	*Z*	1.758	1.723	1.728	1.436	1.363
	*P*	0.079	0.085	0.084	0.151	0.173

As illustrated in Figure 6, the ESV of Shiqu County generally exhibits a spatial distribution pattern with high values in the north and south, and low values in the central part. From the perspective of the area proportion and distribution of each per unit area ESV grade, the medium grade dominates, accounting for approximately 69.99%, primarily distributed in the grassland‐dense zones of the southeastern, central, and northern regions. The low grade constitutes about 17.88%, mainly located in the unused land and grassland transition zones of the southwestern, southeastern, and northern areas. The high‐grade accounts for about 6.97%, predominantly in the forest‐dense mountains of the south. The extremely low grade, at approximately 4.74%, is concentrated in the unused land areas of the southwest, southeast, and north. The extremely high grade, comprising only about 0.42%, is focused around the lakes in the south and north. From 1980 to 2020, the area of per unit area ESV grades showed a trend of expansion in high and extremely high grades, while other grades experienced a reduction. Specifically, the area of high and extremely high grades expanded by 9.96% and 99.73%, respectively, while the extremely low, low, and medium grades decreased by 3.69%, 0.55%, and 0.98%, respectively.

From the perspective of the distribution and changes of per unit area ESV at the township scale (Figure 7), the ESV per unit area at the township scale in Shiqu County presents a pattern of high values in the south and low values in the north. Townships such as Waxu, Qiwu, Wenbo, and Majia in the southeastern region, where forests and water areas are dense, have higher per unit area ESV, whereas in the central and northern regions, where grasslands and unused lands are extensively spread, townships like Gemeng, Gayi, Mengyi, Arizha, and Nijia have lower per unit area ESV. From 1980 to 2020, a total of 12 townships in Shiqu County experienced a decrease in ESV, mainly concentrated in the eastern and western regions, with the per unit area ESV (10^4^ CNY/ha) in Maga, Zhengke, Benda, and Luoxu townships decreasing by 0.05, 0.03, 0.02, and 0.02, respectively. Areas with an increase in per unit area ESV were mainly concentrated in the central‐northern and southeastern regions, with Changshagongma, Xinrong, Gayi, and Gemeng townships experiencing particularly rapid growth, increasing by 0.21, 0.06, 0.02, and 0.01, respectively.

This indicates that the spatial distribution and changes of per unit area ESV in Shiqu County are consistent with the patterns of land‐use distribution and changes: the expansion of unused lands in the eastern, western, and southern border areas has led to a local decrease in ESV; the central‐northern and southeastern regions have seen an increase in local ESV due to the expansion of forest and water areas. Therefore, it is necessary to optimize the land‐use structure according to local conditions to promote the sustainable growth of regional ecosystem services. For example, in areas with high ecological value, such as grasslands or wetlands, policies could prioritize conservation and restoration efforts to enhance ecosystem services like water purification and carbon sequestration. In high‐altitude areas that provide critical ecosystem services such as water regulation and biodiversity support, targeted conservation initiatives can help maintain these essential functions. In mid‐altitude zones where human activity is more concentrated, strategies such as promoting controlled grazing or improving habitat connectivity could be implemented to balance ecological preservation with socioeconomic needs.

Based on the changes in the standard deviational ellipse parameters of ESV in the study area (Table 5), the azimuth angle of ESV fluctuated within the range of 161.590° to 162.432° from 1980 to 2020, showing an overall increasing trend. This indicates an intensifying expansion of ESV from an “east–west” orientation toward a “southeast‐northwest” orientation. The minor axis decreased from 49.778 km in 1980 to 49.194 km in 2020, while the major axis increased from 77.974 km to 79.773 km, suggesting a slight contraction in ESV distribution. This pattern was relatively stable in the east–west direction, with an expanding trend in the north–south direction. The ratio of the major to minor axis rose from 1.566 in 1980 to 1.622 in 2020, indicating an increasing spatial concentration of ESV in the study area.

**TABLE 5:** Changes in standard deviation ellipse and centroid parameters of ESV in the study area.

Year	Standard deviation ellipse				Center of gravity	
	Azimuth angle /°	Major axis/km	Minor axis/km	Oblateness	Longitude (E)/°	Latitude (*N*)/°
1980	161.643	77.974	49.778	1.566	98.211	33.148
1990	161.599	77.960	49.729	1.568	98.210	33.149
2000	161.590	77.964	49.719	1.568	98.210	33.149
2010	162.432	79.782	49.277	1.619	98.204	33.169
2020	162.341	79.773	49.194	1.622	98.205	33.169

**FIGURE 8:**
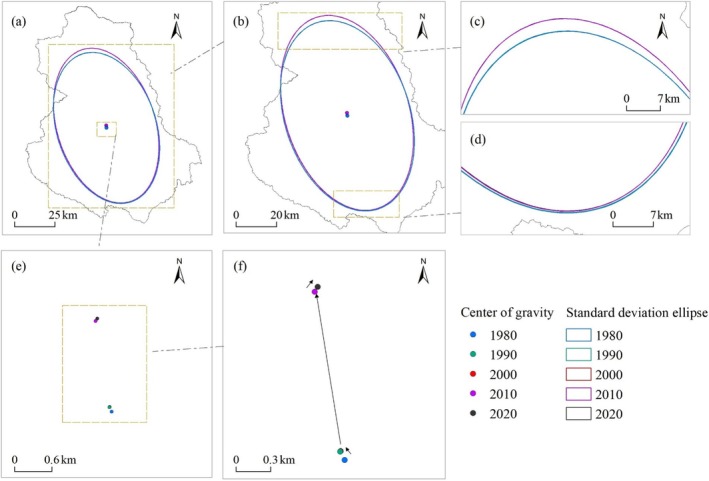
Distribution and changes in the standard deviation ellipse and centroid of ESV in the study area: (a) Overall distribution and changes in the standard deviation ellipse and centroid of ESV; (c) and (d) Enlarged details of the standard deviation ellipse; (e) and (f) Enlarged details of the center of gravity.

Regarding the distribution range and centroid shift of the standard deviational ellipse (Figure 8), the expansion direction of ESV in the study area followed a southeast–northwest trajectory from 1980 to 2020. Except for slight northeastward shifts during 1990–2000 and 2010–2020, the centroid of the ESV standard deviational ellipse moved a total of 2.453 km northwest from 1980 to 2020.

### 3.5 Topographic Gradient Differentiation of Ecosystem Service Values

**FIGURE 9:**
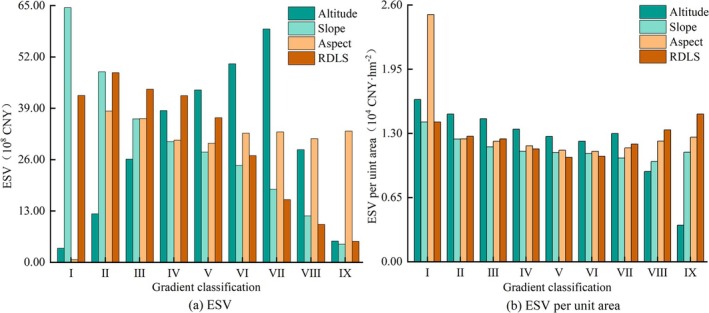
Terrain gradient distribution of ESV in Shiqu County in 2020: (a) Total ESV across various terrain gradients in 2020, and (b) Per‐unit‐area ESV across different terrain gradients in 2020.

Using the year 2020 as a reference, the study analyzed the topographic gradient differentiation of ESV in Shiqu County. Regarding the overall distribution of ESV (Figure 9a), ESV initially increases and then decreases with the increase in altitude and terrain ruggedness. The maximum ESV values for these two topographic gradients are observed at altitude level VII and terrain ruggedness level I, amounting to 5.909 billion CNY and 6.449 billion CNY, respectively. As the slope increases, ESV shows a decreasing trend, with the maximum value of 6.449 billion CNY at slope level I. In terms of aspect, apart from a minimal distribution of 0.070 billion CNY at aspect level I, ESV distributions across other levels are relatively uniform, ranging from 3.017 billion CNY to 3.832 billion CNY, with the highest value at aspect level II (Table S1).

In terms of the distribution of per unit area ESV (10^4^ CNY/ha) across topographic gradients (Figure 9b), per unit area ESV continuously decreases with increasing altitude, with extreme values of 1.64 at altitude level I and 0.37 at level IX. On the slope and terrain ruggedness scales, per unit area ESV shows a trend of decreasing and then increasing, with transition zones at slope level VIII (1.01) and ruggedness level V (1.06), and the maximum values being 1.42 at level I for slope and 1.50 at level IX for ruggedness. On the aspect scale, apart from the highest value of 2.50 at level I, the values across other levels range between 1.12 and 1.26.

**FIGURE 10:**
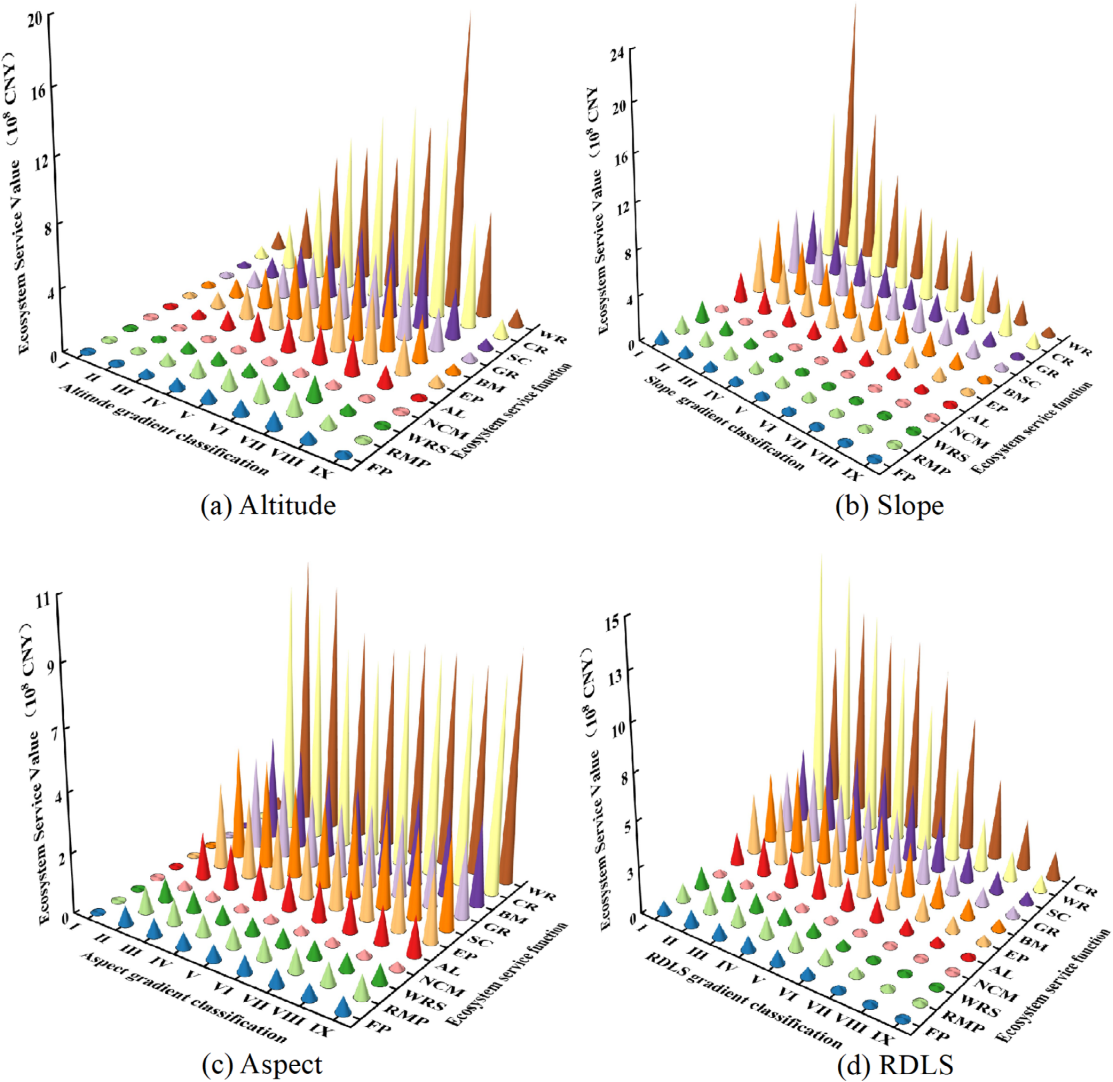
Terrain gradient distribution of ESV for various ecosystem service functions in Shiqu County: (a) ESV distribution across elevation gradients for various ecosystem service functions, (b) ESV distribution across slope gradients for various ecosystem service functions, (c) ESV distribution across aspect gradients for various ecosystem service functions, and (d) ESV distribution across RDLS gradients for various ecosystem service functions. FP: Food production; RMP: Raw material production; WRS: Water resource supply; GR: Gas regulation; CR: Climate regulation; EP: Environment purification; WR: Water regulation; SC: Soil conservation; NCM: Nutrient cycle maintenance; BM: Biodiversity maintenance; AL: Esthetic landscape; TL: Total. The same below.

Further analyzing the year 2020 as a representative year, the study examines the topographic gradient differentiation of various ecological system service functions' ESV (billion CNY) in Shiqu County. On the altitude gradient, the ESV of each ecological system service function shows a pattern of initial increase followed by decrease (Figure 10a), with altitude level VI being the transition zone for Raw Material Production (RMP), Climate Regulation (CR), and Natural Disaster Mitigation (NCM), with respective ESVs of 1.36, 12.87, and 0.44. The transition zones for other ecological system service functions are at level VII, with the ESVs for Food Production (FP), Water Resource Supply (WRS), Gas Regulation (GR), Erosion Prevention (EP), Waste Recycling (WR), Soil Conservation (SC), Biodiversity Maintenance (BM), and Aesthetic Landscape (AL) being1.01, 1.53, 4.80, 4.70, 18.76, 5.83, 5.48, and 2.48 respectively (Table S2).

On the slope gradient, the ESV of each ecological system service function shows a decreasing trend (Figure 10b), with the maximum values all at slope level I. The respective ESVs for FP, RMP, WRS, GR, CR, EP, WR, SC, NCM, BM, and AL are 1.06, 1.41, 1.81, 4.99, 13.16, 4.95, 22.30, 6.03, 0.46, 5.72, and 2.61. On the aspect gradient, the ESV of each ecological system service function, apart from being lower at aspect level I, is relatively uniformly distributed across other levels (Figure 9c), with the maximum values all at aspect level II. The respective ESVs for FP, RMP, WRS, GR, CR, EP, WR, SC, NCM, BM, and AL are 0.69, 0.98, 0.83, 3.43, 9.24, 3.20, 9.90, 4.16, 0.31, 3.85, and 1.72. On the terrain ruggedness gradient, the ESV of WRS and WR decreases with increasing terrain ruggedness, with maximum values of 1.15 and 14.24, respectively. The ESV of EP and AL follows an increasing‐then‐decreasing pattern, with the maximum values at ruggedness level II, being 3.91 and 2.12, respectively. FP, RMP, GR, CR, SC, NCM, and BM show a pattern of rise‐decline‐rise‐decline, with ruggedness levels II and IV being transition zones. The maximum values occur at ruggedness level II, with respective ESVs of 0.85, 1.18, 4.18, 11.04, 5.07, 0.38, and 4.72 (Table S2).

Evidently, as altitude, slope, aspect, and terrain ruggedness increase, the distribution of land use exhibits significant vertical zonality, leading to marked vertical differences in ESV, per unit area ESV, and the ESV of each ecological system service function. Therefore, region‐specific land‐use planning should be formulated in accordance with the actual conditions of each area.

### 3.6 Driving Factors of Spatial Differentiation in Ecosystem Service Values

**FIGURE 11:**
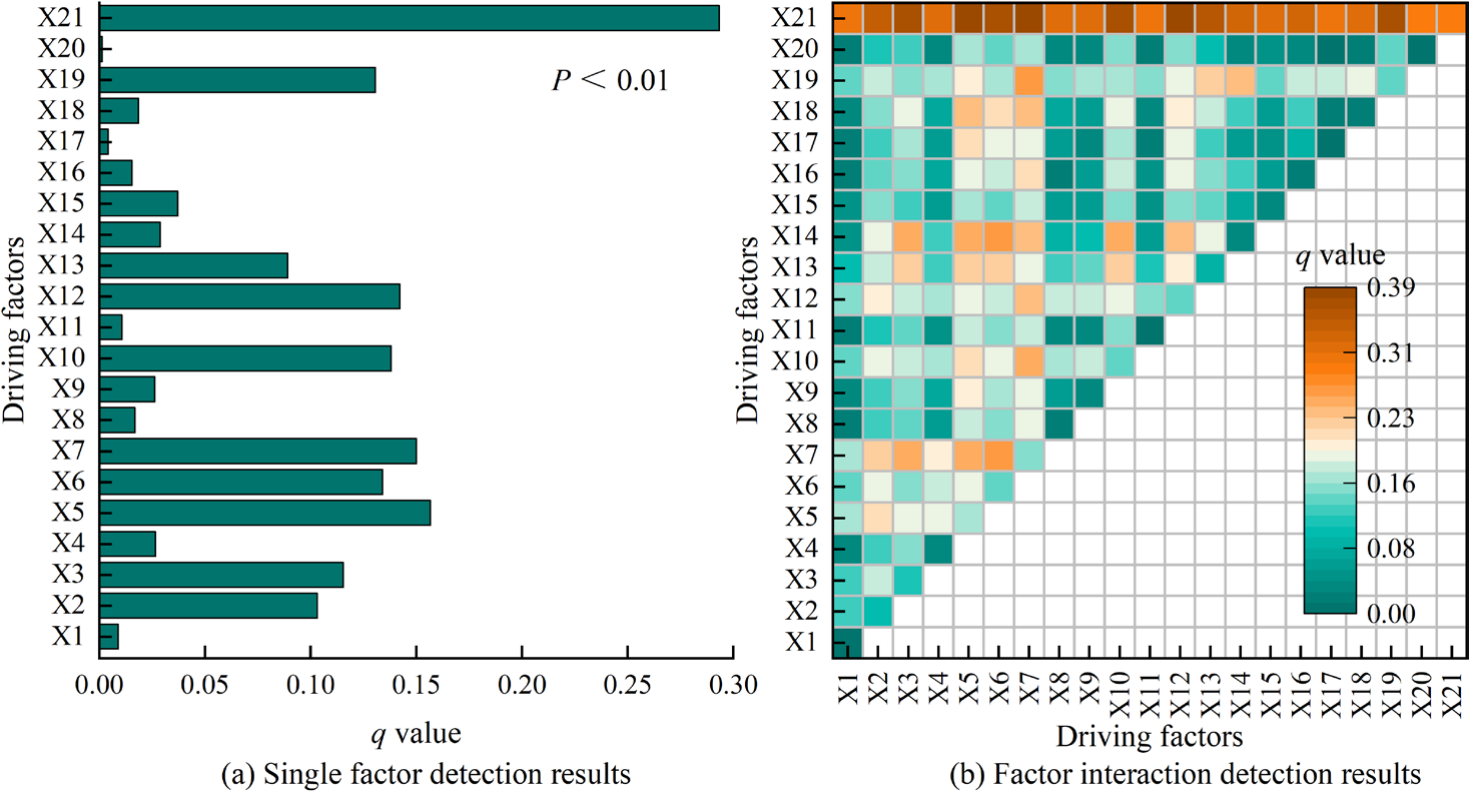
Detection results of driving factors for spatial differentiation of ESV in Shiqu County: (a) *p* values and *q* values of driving factors, and (b) Factor interaction detection results.X1: Agricultural production potential; X2: NDVI; X3: Average annual wind speed; X4: Distance from residential areas; X5: Average annual temperature; X6: Average annual ground temperature; X7: Annual sunshine; X8: Average GDP; X9: Distance from road; X10: Annual evaporation; X11: Distance from rivers; X12: Average annual relative humidity; X13: Distance from reserves; X14: Average annual precipitation; X15: ≥ 10°C accumulated temperature; X16: Population density; X17: Slope; X18: RDLS; X19: Altitude; X20: Aspect; X21: Land‐use types.

To explore the driving mechanisms of spatial differentiation in ESV in Shiqu County, 21 natural and economic factors were selected. The explanatory power of these driving factors, ranked from highest to lowest, is as follows: X21 > X5 > X7 > X12 > X10 > X6 > X9 > X3 > X2 > X13 > X15 > X14 > X4 > X18 > X8 > X16 > X11 > X1 > X17 > X20 (Figure 11a). Among these, X21 is the dominant factor in the spatial differentiation of Shiqu's ESV, with an explanatory power of 0.29. Factors X5, X7, X12, X10, X6, X9, X3, and X2 are relatively important, with explanatory powers of 0.16, 0.15, 0.14, 0.14, 0.13, 0.13, 0.12, and 0.10, respectively. The remaining factors, though with explanatory powers below 0.10, also influence the spatial differentiation of ESV in Shiqu (Table S3).

From the results of the interaction detection among the driving factors (Figure 11b), the impact of any two interacting factors on the spatial differentiation of ESV in Shiqu is greater than that of a single factor. The explanatory power of X21 interacting with other factors is above 0.30, particularly high with X5, X7, and X12 at 0.39, 0.39, and 0.38, respectively. Additionally, interactions like X3∩X7 (0.26), X3∩X14 (0.25), X5∩X7 (0.26), X5∩X14 (0.26), X6∩X7 (0.26), X6∩X14 (0.27), X7∩X10 (0.25), X7∩X19 (0.26), and X10∩X14 (0.25) have notably high explanatory powers, all above 0.25. Interactions between other factors have explanatory powers below 0.25 but still impact the spatial differentiation of ESV. This indicates that the spatial differentiation of ESV in Shiqu County is influenced by both natural and economic factors, with natural factors like land use (X21), annual average temperature (X5), annual sunshine hours (X7), and annual average relative humidity (X12) having a more significant impact on spatial differentiation than economic factors such as distance to protected areas (X13), distance to residential areas (X4), distance to roads (X9), and per capita GDP (X8) (Table S4).

## 4 Discussion

The findings from the study conducted between 1980 and 2020 reveal a decrease in grassland coverage and a simultaneous expansion of construction land and water area in the area. Grasslands, despite their reduction in coverage, remain the most significant contributor to ESV, accounting for approximately 77.96% of the total, emphasizing their pivotal role in maintaining regional ecological balance. This highlights the potential ecological threats arising from rapid changes in land use in the area. Simultaneously, lakes play an indispensable role in hydrological regulation, with their impact on downstream flow ensuring water security and amplifying the contribution rate of ESV in the region (Wang et al. 2023a). These insights provide valuable inputs for the precise implementation of relevant ecosystem management and development strategies (Yang et al. 2023a). It is important to note that the observed changes in land use are likely the result of various factors, including climate change, population growth, economic development, and policy decisions. For instance, climate change can lead to grassland degradation, the shrinking of glacial permafrost, and the expansion of rivers and lakes due to rising temperatures, while increased population and urbanization have driven the expansion of cropland and built‐up areas (Jiang et al. 2020; Yang et al. 2023a; Yang et al. 2022b). The observed changes are shaped by intricate interactions among various drivers, including climate change, policy interventions, and socioeconomic dynamics, which collectively influence land use and ESV trends (Druckenmiller 2022; Yi et al. 2023; Zhang et al. 2023). Since 2000, grassland areas have gradually decreased, likely due to the increasing pressure from grazing activities and the conversion of grasslands into construction land. To mitigate this trend, the “Grassland Ecological Compensation Policy” and the “Restoration of Grazing to Grassland Policy” have been implemented since 2000 (Zhang et al. 2018). The forested area increased steadily from 1980 to 2010, potentially driven by afforestation initiatives and the “Grain‐for‐Green Program” (Wang et al. 2023b). Construction land, on the other hand, expanded significantly after 2000, closely associated with the development of transportation networks and the expansion of residential areas in recent years (Liang and Song 2022). The intricate interactions between land‐use changes and ESV warrant further investigation and underscore the need for effective conservation and sustainable land management strategies (Chen and Liu 2021). To mitigate these potential ecological threats, policymakers should prioritize strategies such as grassland restoration, sustainable water management, and controlled urban expansion to balance ecological preservation with regional development goals.

Throughout the study period, Shiqu County's Ecosystem Service Value (ESV) displayed a continuous growth pattern, increasing from 226.180 billion CNY in 1980 to 26.848 billion CNY in 2020, representing a net increase of 2.55%. Notably, the ESV of water areas exhibited a substantial growth of 33.53%, driven by the expansion of lakes and wetlands, which are increasingly recognized for their hydrological and climate regulation benefits. This transformation from grasslands to water areas in response to climate change has the potential to result in a higher ESV per unit area (He et al. 2022; Wang et al. 2016). Climate regulation and hydrological regulation emerged as significant components, constituting approximately 24.63% and 24.28% of the total ESV, respectively. During the period from 1980 to 2020, hydrological regulation and water supply experienced notable growth, increasing by 9.07% and 8.81%, respectively. This underscores the potential advantages of expanded water surfaces in the Tibetan Plateau due to climate warming. The observed ESV increase may indicate positive trends in ecosystem health and functioning. However, it is essential to recognize that this increase might also mask vulnerabilities within the system, including potential ecological risks arising from extensive grassland reduction (Lyu et al. 2022). They also pose challenges, including the displacement of other land uses and potential alterations to biodiversity. Future research should address these trade‐offs to develop adaptive management strategies.

The ESV of Shiqu County exhibits significant vertical stratification along topographic gradients. Its spatial and temporal distribution is influenced by a range of factors, including both natural and economic elements (Yang et al. 2022). Key factors influencing ESV identified in this study include land use, annual average temperature, annual sunshine hours, and annual relative humidity, with natural factors exerting a dominant influence on shaping the spatial and temporal dynamics of ESV. These factors induce changes in ESV by reshaping the natural landscape and affecting ecosystem structure, processes, and functions (Straton 2006). Furthermore, the interactions between pairs of these factors have a more pronounced impact on spatial ESV variation than individual factors in isolation (Cheng et al. 2022; Su and Fu 2013). The interactions between natural factors, such as terrain gradient and climatic conditions, and socioeconomic drivers highlight the need for an integrated management approach that considers both ecological thresholds and human development demands. Future studies should explore how anthropogenic activities amplify or mitigate these interactions, particularly in regions undergoing rapid socioeconomic transformations, to ensure effective conservation efforts. Alpine ecosystems exhibit heightened vulnerability and irreversibility, making the formulation of scientifically sound ecosystem management policies and conservation efforts crucial for their sustainable development (Kang et al. 2022). Given the observed decrease in grassland and the expansion of construction land, ESV may experience further declines unless sustainable land management practices are implemented. Policies such as the “Grassland Ecological Compensation Policy” and “Returning Grazing Land to Grassland Policy” could play a critical role in mitigating these losses by promoting grassland restoration and reducing degradation caused by overgrazing (Zhang et al. 2018). Future studies should focus on integrating climate change scenarios and socio‐economic projections to develop more comprehensive models for predicting ESV trends (Schirpke and Tasser 2024). This would help refine policy interventions and ensure the sustainable management of ecosystem services in the face of rapid environmental changes.

Areas with high Ecosystem Service Values (ESV) in the studied region are primarily situated within the southern forests and northern wetlands of Shiqu County, both of which are encompassed by Natural Reserves. However, it is worth noting that some high‐value areas are still in proximity to areas of human activity and urban development, where land‐use changes could exacerbate the situation (Yang et al. 2023). Land‐use changes, particularly the transition from grasslands, forests, and wetlands to built‐up areas, may exacerbate the loss of ecosystem service values, such as impacts on water supply, climate regulation, vegetation alteration, disruption of species habitat continuity, and threats to species survival (Liang and Song 2022; Stokstad 2020; Zhou et al. 2024). Furthermore, our results indicate that grasslands and forests at elevations IV–VII, slopes I–II, and EDLS I–IV possess higher biodiversity maintenance capacity. These areas are typically habitats for rare and endangered species, such as the Tibetan Wild Ass ( *Equus kiang* ) and the White‐lipped Deer ( *Cervus albirostris* ), which depend on these high‐altitude grasslands and forest environments (Yang et al. 2024). High‐value water regulation areas are concentrated in wetlands and alpine lake regions at elevations VI–VII, slopes I–II, and EDLS II–IV, which serve as water conservation zones and can effectively regulate both water quantity and quality. Forests and wetlands in the southern part of Shiqu County play a significant role in climate regulation (CR), whereas deforestation, grassland degradation, and urban expansion directly impact the climate regulation capacity of these areas (Gregor et al. 2024). This highlights the urgency of adopting more balanced conservation and development policies to manage the transformation of regional ecosystem service values (He et al. 2022). Simultaneously, it is the responsibility of the government to carefully realign societal demands while ensuring the sustainability of ecosystems amid economic growth (Shi et al. 2021). The expansion of urban areas and development initiatives necessitates a dedicated commitment to mitigating the depletion of natural resources. Therefore, as we benefit from the various services provided by the ecosystem, it is essential to thoughtfully consider the ecological thresholds that enable sustainable ecosystem functioning (Peng et al. 2017). The findings from this study underscore the importance of adaptive policies that align with local ecological characteristics. For example, in high‐ESV areas near human settlements, zoning regulations could minimize land‐use conflicts, while incentive‐based conservation programs may promote sustainable practices. This research framework and its insights can be applied to other fragile ecosystems, such as the Andes or Himalayas, to inform globally relevant conservation and land management strategies.

## 5 Limitations

The use of fixed ESV coefficients, while widely adopted, may oversimplify the dynamic and context‐dependent nature of ecosystem services. Although we conducted a sensitivity analysis to evaluate the robustness of these coefficients, they may not fully capture the dynamic changes in ESV driven by local environmental and socioeconomic variations over time. While the study identifies natural and economic factors as key drivers of ESV spatial differentiation, other potentially influential elements, such as policy interventions, cultural values, or anthropogenic pressures, were not explicitly analyzed, which may limit the comprehensiveness of the findings. The study examines ESV changes over decadal intervals, which may overlook short‐term fluctuations or abrupt changes in ESV due to specific events, such as extreme weather or policy shifts.

## 6 Conclusions

Shiqu County's land use is primarily dominated by grasslands and unused lands, accounting for approximately 77.96% and 12.15% of the total county area, respectively. Over the period from 1980 to 2020, there was an overall trend of decreasing unused land, grassland, and cultivated land, coupled with an expansion of forest land, water areas, and urban areas. Particularly noteworthy were the significant changes in the extents of urban areas, water areas, and cultivated land.

Throughout the study duration, Shiqu County's ESV exhibited a growth trajectory, increasing from 26.180 billion CNY in 1980 to 26.848 billion CNY in 2020, representing a net increase of 2.55% (0.668 billion CNY). Grasslands played a substantial role in contributing to Shiqu's ESV. The ESV of water areas showed remarkable growth, increasing by 33.53%. Climate regulation and hydrological regulation emerged as significant, constituting approximately 24.63% and 24.28% of the total ESV, respectively. From 1980 to 2020, all ecosystem service functions exhibited ESV growth, with hydrological regulation and water supply notably increasing by 9.07% (0.562 billion CNY) and 8.81% (0.046 billion CNY), respectively.

The high‐grade category accounts for approximately 6.97% of the ESV, primarily concentrated in the densely forested mountains of the south. The extremely high grade, comprising only about 0.42%, is concentrated around the lakes in the south and north. Between 1980 and 2020, there was a trend of expansion in high and extremely high‐grade ESV categories, while other grades experienced reductions. Specifically, the area covered by high and extremely high grades expanded by 9.96% and 99.73%, respectively.

With increasing altitude, slope, aspect, and terrain ruggedness, the land‐use distribution exhibits significant vertical zonation, resulting in pronounced vertical variations in ESV, per unit area ESV, and ESV across various ecosystem service functions. The spatial differentiation of ESV in Shiqu County is influenced by both natural and economic factors, with natural factors exerting a more substantial influence on spatial disparities than economic factors. These research findings are significant for guiding land management decisions and policy formulation on the Qinghai‐Tibet Plateau. These findings are useful for stakeholders, local communities or beneficiaries, resource managers, and government agencies to implement policies that consider ecosystem well‐being.

## Author Contributions

**Bin Feng:** data curation (equal), formal analysis (equal), writing – original draft (equal). **Jianwei Zhou:** data curation (equal), formal analysis (equal), writing – original draft (equal). **Lu Hu:** writing – original draft (equal). **Zherong Liu:** data curation (equal). **Yi Yang:** data curation (equal). **Shuai Yang:** data curation (equal). **Jianying Ni:** data curation (equal). **Wenke Bai:** resources (equal), supervision (equal), writing – review and editing (equal). **Shanshan Zhao:** resources (equal), supervision (equal), writing – review and editing (equal).

## Conflicts of Interest

The authors declare no conflicts of interest.

## Supporting information


Appendix S1.

Appendix S2.

Appendix S3.

Appendix S4.

Appendix S5.

Table S1.

Table S2.

Table S3.

Table S4.

## Data Availability

Related data are openly available in Dryad at https://doi.org/10.5061/dryad.0cfxpnw93.
